# Update Nierentransplantationspathologie: Eine bessere Darstellung der mikrovaskulären Entzündung

**DOI:** 10.1007/s00292-024-01328-3

**Published:** 2024-04-22

**Authors:** Nicolas Kozakowski

**Affiliations:** Klinisches Institut für Pathologie, Medizinische Universität Wien, Währinger Gürtel 18–20, 1090 Wien, Österreich

**Keywords:** Antikörper, Abstoßung, Diagnostische Empfehlungen, Genexpressionsanalyse, Nierentransplantatbiopsie, Antibody, Rejection, Diagnostic recommendations, Gene expression analysis, Kidney transplant biopsy

## Abstract

**Hintergrund:**

Die Banff Foundation erzeugt Empfehlungen für die pathologische Klassifikation variabler Läsionen der Nierentransplantatabstoßung. Alle 2 Jahre versammeln sich Experten, um die Empfehlungen anhand neuer wissenschaftlicher und klinischer Erkenntnisse zu aktualisieren.

**Ziel der Arbeit:**

Dieser Beitrag stellt die wichtigsten Änderungen der letzten Banff-Konferenz vor.

**Material und Methoden:**

Der Autor dieses Beitrags nahm persönlich an der Banff-Konferenz und der darauffolgenden Umfrage teil, berichtete über die Aktivitäten einer Banff-Arbeitsgruppe (peritubuläre Kapillaritis) vor Ort und wirkte an der Abfassung des rezent veröffentlichen Meeting-Reportes mit.

**Ergebnisse:**

Läsionen der antikörpermediierten Abstoßung (AMR) in Nierentransplantaten und insbesondere die mikrovaskuläre Entzündung sind seit über 20 Jahren Teil des diagnostischen Algorithmus. Ein vereinfachter Algorithmus der AMR und ein vorsichtiger Einschluss molekularpathologischer Daten in die klinisch-pathologische Beurteilung und Therapieentscheidung wurden befürwortet. Eine neue diagnostische Entität – mikrovaskuläre Entzündung, C4d-negativ und donorspezifische Antikörper negativ – wurde in die Kategorie der AMR eingeführt, um diesen pathophysiologisch und immunologisch wahrscheinlich andersartigen Phänotyp anzuerkennen und Forschung hierauf zu motivieren.

**Schlussfolgerung:**

Die Banff-Klassifikation bietet eine Struktur für die Befundung in der Nierentransplantatpathologie. Regelmäßige Aktualisierungen dienen der Anpassung an stets wachsendes Wissen. Besondere Herausforderungen sind dabei, die Komplexität verschiedener immunologischer Situationen zu erfassen und eine verständliche Abbildung davon in einem pathologischen Befund zu gewährleisten.

## Abstract (en)

**Background:**

The Banff Foundation produces recommendations for classifying various lesions in renal allografts. Experts gather to update the classification every other year based on new scientific and clinical evidence.

**Objectives:**

This article presents the most important changes incorporated into the new recommendations after the last Banff conference.

**Materials and methods:**

The author of this article personally took part in the Banff conference and the subsequent survey, reported on the activities of a Banff working group (peritubular capillaritis) on-site, and contributed to drafting the recently published meeting report.

**Results:**

Lesions of antibody-mediated kidney allograft rejection (AMR), especially microvascular inflammation, have been part of the diagnostic algorithm for over 20 years. Experts advocated for a simplified AMR algorithm and mindful inclusion of molecular pathological data in the clinicopathological reflection regarding therapeutic decision. A new, more descriptive diagnostic entity—microvascular inflammation, C4d negative and DSA negative—has been introduced into the AMR category to acknowledge this histological constellation and motivate research into this pathophysiologically and immunologically probably different phenotype.

**Conclusions:**

The Banff classification provides a structure for diagnosing kidney transplant pathology. Regular updates serve to adapt to ever-growing knowledge about alloimmunity. Particular challenges are capturing the complexity of various immunological scenarios and ensuring an understandable representation of these in a pathology report.

Die letzte Tagung der Banff-Klassifikation für die Pathologie der Nierentransplantatabstoßung kehrte im September 2022 nach Banff, Alberta, Kanada zurück. Anlässlich ihres 30. Geburtstags wurden vergangene Erfolge und zukünftige Aufgaben der „Banff Foundation“ im Rahmen eines Pre-Meetings besprochen. Basierend auf rezenten Studien hat sich die Bewertung der Läsionen der antikörpervermittelten Abstoßung („antibody-mediated rejection“, AMR) – und der Einsatz molekularpathologischer Techniken als aktualisierungswürdig herauskristallisiert [16].

## Mikrovaskuläre Entzündung neu definiert

Die Banff-Klassifikation gliederte sich noch bis vor dem letzten Update wie folgt:

- Kategorie 1: normaler Befund bzw. unspezifische Veränderungen,
- Kategorie 2: antikörpermediierte Abstoßung,
- Kategorie 3: suspekt für T‑Zellen-mediierte Abstoßung (Borderline-Läsionen),
- Kategorie 4: T‑Zellen-mediierte Abstoßung,
- Kategorie 5: interstitielle Fibrose und Tubulusatrophie,
- Kategorie 6: andere Veränderungen ohne Zusammenhang mit einer Abstoßungsreaktion (z. B. Polyomavirusnephropathie, lymphoproliferative Post-Transplantat-Erkrankung, Calcineurin-Inhibitorentoxizität, akuter Tubulusschaden, Glomerulonephritis, Pyelonephritis etc.).

Über 1100 Nierentransplantatbiopsien (hiervon zwei Drittel Indikationsbiopsien und ein Drittel Protokollbiopsien), entnommen zwischen 2020 und 2023 in unserem Zentrum, zeigten folgende Inzidenzen gemäß der Banff-Klassifikation 2019: Borderline-Läsionen (4 %), T‑Zellen-mediierte Abstoßung (3 %), antikörpermediierte Abstoßung (13 %), thrombotische Mikroangiopathie (3 %), Polyomavirusinfektion (3 %) und normale Histologie oder interstitielle Fibrose und Tubulusatrophie oder andere non-alloimmune Pathologie (74 %). Wie darunter erklärt, ist diese pathophysiologienahe Klassifizierung nicht exklusiv, da mehrere Phänotypen gleichzeitig vorkommen können, und auch komplex, da manche Läsionen verschiedenen Phänotypen angehören können und inkomplette Phänotypen beinhalten.

## Entwicklung der AMR-Diagnostik

Seit den Frühzeiten der Transplantation stellen gegen Spenderantigene gerichtete Antikörper – auch donorspezifische Antikörper (DSA) genannt – bekanntlich eine ernste Gefahr für transplantierte Nieren dar. Ab den späten 1960er-Jahren wurde daher zur Identifikation von Patienten mit DSA die sog. Cross-match-Untersuchung verwendet. Ziel war die Vermeidung der hyperakuten Abstoßung, für die es keine therapeutische Option gab. Die Rolle von (De-novo‑)DSA zu späteren Zeitpunkten nach Transplantation fand wenig Aufmerksamkeit, obwohl manche Arbeitsgruppen bereits in den frühen 1990er-Jahren die Assoziation mikrovaskulärer leukozytärer Infiltration, einer oft granulozytären Inflammation in den Glomerula oder den peritubulären Kapillaren, mit der Detektion zirkulierender DSA, und einem schlechten Verlauf nachweisen konnten [22].

Der damals technisch sehr schwierige Nachweis von DSA verhinderte die Etablierung der AMR-Diagnostik in der klinischen Praxis. Erst der immunhistologische Nachweis mikrovaskulärer Ablagerungen des Komplementspaltprodukts C4d in Transplantatbiopsien erlaubte es, mit technisch einfachen Mitteln eine durch DSA vermittelte Aktivierung des Komplementsystems darzulegen. An diese Entwicklung war unser Zentrum Anfang der 2000er-Jahre aktiv beteiligt [3]. Für die Diagnose der AMR definierte man dann die diagnostische Kriterientriade: 1 – histologisch sichtbare Gewebeläsionen der AMR, 2 – positiver Nachweis der Komplementsystemaktivierung und 3 – Detektion von DSA.

**Abb. 1:**
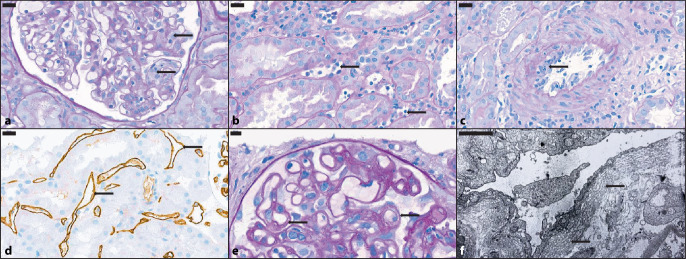
Pathologische Läsionen der antikörpermediierten Abstoßung: **a** Glomerulitis („g“) mit okklusiver endokapillärer Hyperzellularität (*schwarze Pfeile*) durch Leukozyten und aktivierten Endothelien. Das einfache Vorhandensein von intraluminalen Leukozyten ist für diese Läsion nicht ausreichend. Indirekte Zeichen einer Interaktion mit dem Endothel, wie z. B. eine Schwellung der Endothelien, und eine Einengung des Lumens sind erforderlich. Die Läsion wird nach der Proportion der in der Biopsie vorhandenen Glomerula bestimmt, die solche segmentalen oder globalen Läsionen vorweisen (PAS [„periodic acid Schiff reaction“], Maßstabsleiste 20 μm). **b** Peritubuläre Kapillaritis („ptc“) mit ausgeweiteten Lichtungen, infiltrierenden Leukozyten und prominenten Endothelien (*schwarze Pfeile*). Peritubuläre Kapillaren sind definitionsgemäß, und im Gegenteil zu den Vasa recta des Nierenmarks, die in der Nierenrinde zu findenden Kapillaren. Wenn > 10 % der kortikalen peritubulären Kapillaren von Leukozyten infiltriert sind und je nachdem wie viele intraluminale Leukozyten im am schwersten betroffenen peritubulären Kapillar zu detektieren sind, wird der ptc-Score angegeben. Aus der Evaluation ausgeschlossen werden Herde einer Pyelonephritis oder eines Infarktes sowie unmittelbar subkapsuläre Entzündungen. Peritubuläre Kapillaren innerhalb interstitieller Fibrose und Tubulusatrophie sollten, im Gegenteil, mitgerechnet werden. Acht muss auch geboten werden, dass die Evaluation durch die Betrachtung längst geschnittener Kapillaren nicht überschätzt wird. Nur quer geschnittene peritubuläre Kapillaren sollen in dem Scoring herangezogen werden. Über zusätzliche Informationen, v. a. das Ausmaß der Läsion (10–50 % der Nierenrinde: fokal; > 50 %: diffus), die leukozytäre Zusammensetzung (v. a. granulozytär dominiert) und der Grad der kapillären Ausweitung, kann auch berichtet werden (PAS, Maßstabsleiste 20 μm). **c** Intimaarteritis („v“) mit Intimaödem und subendothelial infiltrierenden Leukozyten in einer kleinen intrarenalen Arterie (*schwarze Pfeile*). Eine Arterie sollte mindestens zwei Schichten glatter Muskelzellen in der Tunica media zeigen. Das Ausmaß der entzündlichen Lumeneinengung bzw. eine Gefäßwandnekrose bestimmen den Grad der Läsion (PAS, Maßstabsleiste 20 μm). **d** Positive Immunhistochemie für C4d mit komplettem, linearem, braunem Signal an der Oberfläche der Endothelien der peritubulären Kapillaren (*schwarze Pfeile*, DAB [Diaminobenzidin], Maßstabsleiste 20 μm). **e** Transplantatglomerulopathie („cg“) mit dem typischen Umbau der glomerulären Basalmembranen mit Doppelkonturen (*schwarze Pfeile*). Der am schwersten betroffene Glomerulum (Proportion der Kapillaren des Gefäßknäuels) bestimmt den Score. Diese Läsion kann gelegentlich auch erst in der elektronenmikroskopischen Untersuchung entdeckt werden. Andere Erkrankungen, die zu einem Umbau der Basalmembranen führen können (z. B. eine Glomerulonephritis oder eine thrombotische Mikroangiopathie), sollten ausgeschlossen werden (PAS, Maßstabsleiste 20 μm). **f** Schwere (> 7 Schichte) Aufsplitterung der Basalmembran des peritubulären Kapillaren (Elektronenmikroskopie, Maßstabsleiste 2 μm). Die Banff Foundation hat ein Referenzdokument für diese Läsion produziert [19]

1. Histologische Läsionen der AMR betreffen primär das Kapillarbett des Transplantats (Abb. 1). Dies sind einerseits die glomerulären Kapillarschlingen, andererseits das dichte Netzwerk der sog. peritubulären Kapillaren. Während einer aktiven AMR akkumulieren sich Leukozyten in Kapillaren. Diese werden vor Ort durch die Interaktion zwischen Alloantikörper und dem Empfängerendothel und die daraus resultierende Zytokinenausschüttung und Komplementaktivierung angelockt. Dabei ist nicht nur die intrakapilläre Vermehrung der Immunzellen, sondern auch deren sichtbare Interaktion mit der Kapillarwand (Lumenokklusion und Aktivierung von Endothelzellen) diagnostisch relevant. Bei Erfüllung dieser Kriterien in Glomerula und Absenz einer Glomerulonephritis, die ähnlich aussehen könnte, heißt diese Läsion Glomerulitis (Abb. 1a). Die analoge Veränderung in den interstitiellen Kapillaren heißt peritubuläre Kapillaritis (Abb. 1b). Eine Persistenz der Kapillaritis kann einen Umbau der Kapillarwände verursachen, der als Beweis einer langdauernden (chronischen) AMR gesehen wird. Dieser durch Verbreiterung und Aufsplitterung der Basalmembranen gekennzeichnete Prozess führt in den Glomerula zu lichtmikroskopisch sichtbaren sog. Basalmembrandoppelkonturen (Abb. 1e). Frühe Läsionen dieses Umbaus können bereits nach einem Monat detektierbar sein [23]. Eine ähnliche Aufsplitterung und Verbreiterung der Basalmembranen der peritubulären Kapillaren ist allerdings nur elektronenmikroskopisch nachweisbar (Abb. 1f).
2. Die immunhistochemische bzw. mittels Immunfluoreszenz erreichbare Detektion einer Komplementaktivierung über den Nachweis des Komplementspaltprodukts C4d entlang des Endothels der peritubulären Kapillaren (Abb. 1d) gilt als Beweis der Interaktion zwischen Antikörper und Endothel.
3. Der Nachweis von DSA in der Zirkulation des Empfängers spielte eine zentrale Rolle und erfolgte früher ausschließlich über zellbasierte Methoden. Ab den 1960er-Jahren konnte das Empfängerserum mit Lymphozyten potenzieller Spender inkubiert und die Komplement-abhängige Zelllyse („complement-dependent cytotoxicity crossmatch“) untersucht werden. Diese Untersuchung wird in vielen Zentren verwendet und kann, je nachdem, welche Zellsubtypen lysiert werden, die Spezifität der Anti-HLA-Klasse („human leukocyte antigen“) I oder II Alloantikörper bestimmen. In vielen anderen Zentren wird mittlerweile der „virtual crossmatch“ angewandt. In den 1980er-Jahren konnte die Bindung dieser Alloantikörper an Spenderlymphozyten durchflusszytometrisch detektiert werden („flow cytometry crossmatch“). In den 1990er-Jahren wurden ELISA („enzyme-linked immunosorbent assays“)-Untersuchungen eingesetzt, die HLA-spezifische Antikörper im Serum des Empfängers in Well-Platten mit prädefinierten HLA-Antigenen detektieren können. Auch in dieser Zeit wurde die Technologie der „microbeads“ (Mikrokugeln) entwickelt. Diese verwendet heutzutage synthetisch erzeugte HLA-spezifische Antigene, die die Oberfläche der „microbeads“ umhüllen. Die Bindung zirkulierender Alloantikörper wird mittels Immunofluoreszenz durchflusszytometrisch dargestellt und quantifiziert. Eine Ergänzung dieser Technik hat mittlerweile in vielen Zentren großes Ansehen getroffen: die Luminex-Plattform, die die Detektion zunächst der HLA-Spezifität der einzelnen „microbeads“ und unmittelbar danach einer eventuellen Alloantikörperbildung ermöglicht. Die Stärke der Fluoreszenz der einzelnen Spezifitäten wird als mittlere Fluoreszenzintensität (MFI) ausgedrückt. In 2017 wurde C4d als Surrogate für DSA-Positivität (Kriterium 3) akzeptiert [19].

### Zentrale Rolle der mikrovaskulären Entzündung

Im Rahmen der Banff-Konferenz werden Erkenntnisse zur Pathophysiologie der AMR und deren Auswirkungen auf das transplantierte Organ bei den 2‑jährig wiederkehrenden Meetings diskutiert. Technologische Fortschritte erlaubten es Anfang der 2000er-Jahren, die molekularen Hintergründe der Gewebeschädigung durch Abstoßung und insbesondere die zentrale Bedeutung der mikrovaskulären Inflammation für die AMR wahrzunehmen. Es erweiterte sich das pathophysiologische Verständnis der Abstoßungsmechanismen dank Transkriptionsprofilanalysen, wegweisend vom Team um Phil Halloran an der Universität Alberta, Edmonton, Kanada geleitet. Mittels DNA-Microarrays hat das Team versucht, die Modifikationen von Endothelien und Leukozyten als Hauptakteure der Abstoßung, aber auch von anderen renalen Zellen wie Tubulusepithelien und Fibroblasten auf Molekularebene abzubilden. So wurden in histologisch gut definierten AMR-Fällen spezifische Veränderungen auf transkriptioneller Ebene („pathology based transcript sets“) identifiziert. Diese Variationen hoben die zentrale Rolle der Endothelzellen und der natürlichen Killer (NK)-Zellen-vermittelten Immunreaktionen, aber auch der typischen Gewebeschädigung und Reparatur sowie Interferon-Gamma (IFN-γ)-induzierbaren Immunreaktionen im Rahmen der AMR hervor. Diese Entwicklungen werden sehr schön in einer rezenten Review von Halloran et al. [10] dargestellt. Darauffolgend Anfang der 2010er-Jahren wurden die überzeugenden molekularpathologischen Informationen dieser Technologie als ergänzende Parameter zur Abgrenzung verschiedener Abstoßungstypen in die Banff-Klassifikation aufgenommen.

Parallel dazu, als andere Arbeitsgruppen Zugang zu diesen Genexpressionsanalysen bekamen, erlaubten Molekularstudien einerseits die Assoziation mikrovaskulärer Läsionen (g, ptc) mit DSA in multiplen Kohorten zu bestätigen und andererseits zu zeigen, dass AMR-typische Signaturen auch in C4d-negativen Biopsien detektierbar und mit einem schlechteren Transplantatüberleben assoziiert waren [7]. Auf diesen letzten Beobachtungen basiert das Konzept der C4d-negativen AMR und veränderte folglich die Gewichtung diagnostischer Kriterien. Nicht mehr C4d, sondern die mikrovaskuläre Entzündung wurde zum zentralen diagnostischen Kriterium, da diese offenbar mit höherer Sensitivität DSA und AMR anzeigten [20]. Daraus resultierte die Einführung des sog. „microvascular inflammation“ (MVI)-Scores, als Addition zu Glomerulitis („g“-) und peritubulärer Kapillaritis („ptc“-)Scores (MVI = g + ptc).

Abgesehen von Läsionen der Mikrovaskularisation haben mittlerweile auch Arterien eine Rolle in der AMR-Diagnostik. Die zunächst als T‑Zellen-spezifisch angesehene Intimaarteritis (die „v“-Läsion der Banff-Klassifikation) ist als potenziell AMR-assoziierte Läsion erkannt. Grundlage hierfür war die Assoziation vieler vaskulärer Abstoßungen mit HLA-Antikörpern und einem schlechteren Verlauf [14].

### Die Komplexität der Banff Klassifikation

**Abb. 2:**
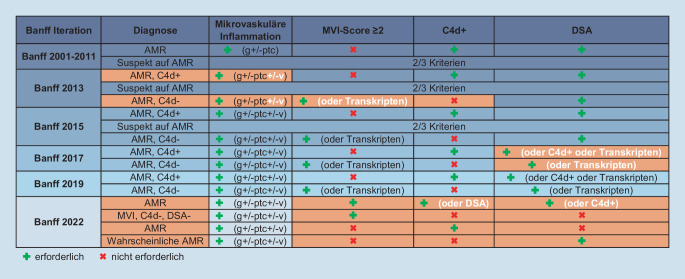
Evolution der AMR-Diagnostik in der Banff-Klassifikation – in *weiß* geschrieben bzw. in den *orangefarbenen* Zellen die Veränderungen im Vergleich zu vorherigen Iterationen (*DSA* donorspezifische Antikörper, *AMR* antikörpermediierte Abstoßung, *MVI* mikrovaskuläre Entzündung, *g* Glomerulitis, *ptc* peritubuläre Kapillaritis, *v* Intimaarteritis, N.B. thrombotische Mikroangiopathie kann u. U., i.e. ohne andere Ätiologie, auch als mikrovaskuläre Entzündung gelten)

Im Laufe der Jahre führte die systematische Analyse der etablierten pathologischen Kriterien der AMR zur Identifikation weiterer inkompletter Befundkonstellationen, wie dem Phänomen von C4d-Ablagerungen ohne weitere morphologische Läsionen. Ein Bild, das typisch bei AB0-inkompatibel transplantierten Nieren zu beobachten war, nicht mit einer Dysfunktion einherzugehen schien und daher als Zustand einer „Akkommodation“ betrachtet wurde. Des Weiteren wurde für eine MVI unterhalb des diagnostischen Schwellenwerts die diagnostische Kategorie „verdächtig auf AMR“ etabliert oder das histologische Bild einer Abstoßung ohne assoziierte Transplantatdysfunktion als subklinische Abstoßung definiert. Diese zunehmend komplizierte Struktur der AMR-Klassifikation reflektierte zwar die Komplexität der Alloimmunität und die Vielfalt der Befundkonstellationen, stieß aber aufgrund der resultierenden Unübersichtlichkeit auf Kritik [10, 11, 15, 17]. Im Bestreben, die Verwirrung durch diagnostisch unklare und fraglich therapeutisch-relevante Diagnosen zu minimieren, wurde auf der Banff-Konferenz 2017 die Kategorie „verdächtig auf AMR“ aus dem Klassifikationsschema eliminiert (Abb. 2).

Die Auswirkungen von Modifikationen der diagnostischen Kriterien auf die Diagnose der ABMR waren der Gegenstand einer eleganten Studie von Callemeyn et al. [4]. Dafür wurden 3662 Biopsien von 949 Nierentransplantaten nach den Definitionen der Banff-Klassifikationen der Jahre 2001, 2013 und 2017 kategorisiert und die Änderungen der Diagnose sowie mögliche Auswirkungen auf den Verlauf analysiert. Man sah, dass die Zahl der AMR-Fälle und Fälle „verdächtig auf AMR“ (sAMR) bei Anwendung der Banff-2013- gegenüber der Banff-2001-Definitionen deutlich zunahm und mit der Anwendung der Banff-2017-Regeln wieder. Von insgesamt 298 sAMR-Fällen gemäß Banff-2013-Kriterien wurden 244 nach den Banff-2017-Kriterien der Kategorie „keine AMR“ zugeordnet. Diese zeigten jedoch verglichen mit „keine AMR“ Fällen nach Banff 2013 ein reduziertes Transplantatüberleben. Die häufigste Befundkonstellation der reklassifizierten Fälle war MVI ohne Nachweis von DSA. Die Autoren schlossen aus ihren Beobachtungen, dass die diagnostische Trennschärfe der AMR-Definitionen zwischen Banff 2013 und Banff 2017 abgenommen hat und MVI-Fälle ohne DSA nicht ohne Weiteres der „keine ABMR“-Gruppe zugeordnet werden sollten. Weitere Studien zeigten eine Assoziation der MVI ohne DSA mit weniger C4d-Ablagerungen, einer recht wechselhaften inflammatorischen Last und variablen Verläufen [6, 13]. Eine immunologisch korrekte und damit klinisch hilfreiche Kategorisierung dieser Fälle kann jedoch nur über das Verständnis der zugrunde liegenden Pathomechanismen erzielt werden.

## Erklärungsmodelle für MVI ohne DSA

Durch die breitflächige Verfügbarkeit der hochsensiblen Luminex-Technik zur Detektion von DSA fiel auf, dass MVI auch ohne nachweisbare zirkulierende Antikörper in Nierentransplantatbiopsien auftreten kann. Prinzipiell kann diese Befundkonstellation auf mindesten 3 unterschiedlichen Umständen beruhen.

1. HLA-DSA sind vorhanden, können aber nicht in der Zirkulation nachgewiesen werden (technische Probleme beim Nachweis, Lücken bei der Spendertypisierung, Absorption der DSA im Transplantat usw.).
2. DSA sind vorhanden, jedoch nicht gegen HLA gerichtet. Derartige Antikörper sind mittels aktueller Luminex-Technologie nicht detektierbar.
3. Die MVI wird tatsächlich durch von Antikörpern unabhängige Mechanismen verursacht.

Vor allem die letzte Option verlangt besondere Aufmerksamkeit, da hier keine Beteiligung von Antikörpern postuliert wird und folglich primär gegen Antikörper gerichtete Therapieformen, die in den beiden anderen Erklärungsmodellen durchaus wirksam sein könnten, nicht zielführend sein werden.

Dieselbe Gruppe konnte in folgenden Arbeiten zeigen, dass alle Fälle mit MVI vergleichbare transkriptionelle AMR-Signaturen unabhängig vom DSA-Status aufwiesen, jedoch DSA-positive MVI-Fälle eine schlechtere Prognose haben. Dies sprach für unterschiedliche pathophysiologische Prozesse bei gleichartigem histologischem Läsionsmuster. MVI-spezifische Transkripte waren u. a. mit IFN-γ-induzierten Pathways, NK-Zellen-Aktivierung und endothelialer Physiologie assoziiert [5]. Die Präsenz von NK-Zellen bei MVI ohne DSA war von besonderem Interesse, da NK-Zellen ursprünglich als Effektoren einer AMR im Sinne einer „antibody-dependent celllular cytotoxicity“ (ADCC) gesehen wurden [2].

Da das Konzept der ADCC auf dem Vorhandensein von gebundenen Antikörpern beruht, könnte bei DSA-negativer MVI ein anderer Mechanismus für die potenzielle Effektorrolle der NK-Zellen vorliegen. Erste Erkenntnisse um die NK-Zellen-Physiologie stammen aus der Tumorimmunologie, da diese spezialisierten Immunzellen eine spontane Zytotoxizität gegen abnormale Zellen und insbesondere Krebszellen aufweisen. Nahezu alle Zellen sollen Moleküle des Haupthistokompatibilitätskomplexes (MHC) Klasse I zeigen, welche als Inhibitor einer unangebrachten NK-Zell-vermittelten Lyse dienen soll. Krebszellen erwerben genetische Veränderung, die u. U. die Struktur oder die Synthese der MHC-Moleküle beeinträchtigen können. Dabei wurde das Konzept des sog. „Missing-Self“ postuliert: bei verminderter Expression oder Verlust solcher Moleküle an der Zelloberfläche fallen die Selbsterkennung und folglich die natürliche Hemmung der NK-Zellen aus. Die lytische Aktivität der NK-Zellen wird genauso in verschiedenen Immunprozessen durch die Erkennung HLA-Moleküle gehemmt. Sollten NK-Zellen jedoch kein bekanntes HLA-Molekül mittels ihrer sog. „Killer Ig-like“ (KIR)-Rezeptoren an der Zelloberfläche vorfinden, wird die Zelllyse eingeleitet, Zytokine ausgeschüttet und andere Leukozyten angelockt. In Analogie zu Krebszellen mit fehlendem Selbst, wurde die Hypothese formuliert, dass NK-Zellen die Zellen eines transplantierten Organs mit abweichenden MHC-Molekülen nicht wiedererkennen und eine lokale Entzündung auslösen. Endothelzellen in der Mikrovaskularisation einer transplantierten Niere stellen die erste Struktur im Kontakt mit dem Immunsystem des Empfängers dar. Aus diesem Grund wurde gedacht, dass der Phänotyp der MVI ohne DSA zumindest teilweise dem „Missing-self“-Mechanismus zugrunde liegen könnte. Rege und überzeugende Forschung um dieses Thema wurde während des letzten Banff-Meetings auch präsentiert.

## Aktualisierung der AMR-Kategorie

Diskussionen während des Meetings sowie eine Online-Konsensbefragung von Experten legten die oben genannten diagnostischen Schwächen der Banff-Klassifikation von 2017 und die oben angeführten neuen pathophysiologischen Erkenntnisse offen. Dies trug zur Entscheidung der Reform der diagnostischen Kategorie 2 (AMR) in der Banff-Klassifikation 2022 bei, mit den Zielen, die AMR-Phänotypen einfacher darzustellen und das Unwissen um den Phänotyp „MVI ohne nachweisbare DSA“ anzuerkennen.

**Abb. 3:**
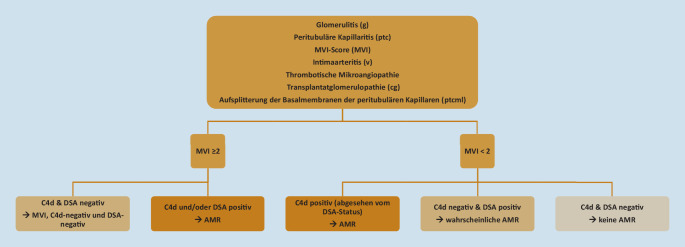
Algorithmus der Kategorie „antikörpermediierte Abstoßung und mikrovaskuläre Entzündung/Schädigung“ entsprechend der Banff-Klassifikation 2022 (*MVI* „microvascular inflammation“, *DSA* donorspezifischer Antikörper, *AMR* antikörpermediierte Abstoßung). (Eigene Darstellung)

Laut neuem Algorithmus sollte zunächst die morphologische Evaluation der Nierentransplantatbiopsie auf Präsenz von AMR-typischen Läsionen erfolgen (Abb. 3). Glomerulitis (g), peritubuläre Kapillaritis (ptc), ihr zusammenfassender MVI-Score (MVI), Intimaarteritis (v), thrombotische Mikroangiopathie ohne andere Ursache, Transplantatglomerulopathie (cg) und die ausschließlich ultrastrukturell detektierbare Aufsplitterung der Basalmembranen der peritubulären Kapillaren (ptcml) zählen zu den Läsionen, die im Rahmen der AMR auftreten können. Je nachdem wie viele Läsionen gefunden werden und wie stark diese ausgeprägt sind, werden folgende diagnostischen Kategorien bzw. Phänotypen definiert:

- *AMR:*
  - Wenn MVI (g + ptc) gleich oder größer als der Schwellenwert von 2 ist, und C4d und/oder DSA nachweisbar sind.
  - Wenn MVI unter dem Schwellenwert von 2 liegt (oder bei v > 0 oder thrombotischer Mikroangiopathie ohne andere Ätiologie) und C4d positiv ist – unabhängig vom DSA-Status.
- *Wahrscheinliche AMR:*
  - Wenn MVI unter dem Schwellenwert von 2 liegt (oder bei v > 0 oder thrombotischer Mikroangiopathie ohne andere Ätiologie), C4d negativ ist und DSA nachweisbar ist.
- *MVI, C4d-negativ, DSA-negativ:*
  - Wenn MVI (g + ptc) gleich oder größer als der Schwellenwert von 2 ist und weder C4d noch DSA nachweisbar sind.
- *Keine ABMR:*
  - Wenn MVI unter dem Schwellenwert von 2 liegt (oder v > 0 oder thrombotische TMA ohne andere Ätiologie) und weder C4d noch DSA nachweisbar sind.

Um die Aktivität und/oder Chronizität der Entzündung zu erfassen, können die oben angeführten Kategorien weiter charakterisiert werden, mit:

- aktiv:
  - wenn keine „cg“- oder „ptcml“-Läsionen nachweisbar sind.
- chronisch-aktiv:
  - wenn „cg“- oder „ptcml“-Läsionen zusätzlich nachweisbar sind.
- chronisch:
  - wenn ausschließlich „cg“- oder „ptcml“-Läsionen nachweisbar sind – kann bei wahrscheinlicher AMR als chronisch-inaktive AMR beschrieben werden.

## Weitere Features der AMR

Während der Konferenz wurden auch folgende Themen um die AMR angesprochen.

Weitere Studien zur Abklärung und Prognoseschätzung der Phänotypen „MVI, C4d-negativ und DSA-negativ“ und „wahrscheinliche AMR“ sind notwendig, und die Arbeitsgruppe AMR – vormals „sensibilisiert“ – sollte dies vorantreiben.

Peritubuläre Kapillaritis und thrombotische Mikroangiopathie (TMA): diesen zwei AMR-assoziierten Einzelläsionen sind eigene Arbeitsgruppen gewidmet. Der Autor dieses Beitrags hat die Ehre, in Beiden mit internationalen Kollegen den Vorsitz zu teilen und berichtete während des Meetings über die Aktivitäten der ptc-Arbeitsgruppe, einer internationalen multizentrischen Studie, um die histologischen Feinheiten dieser Läsion [18]. Die Arbeitsgruppe um die TMA im Transplantat hat kürzlich den ersten Teil einer Konsensbildung über die diagnostischen Kriterien dieser Entität veröffentlicht [1].

In klinischer Praxis sind der aktuelle DSA-Status zum Zeitpunkt der Nierentransplantatbiopsie den Pathologen bzw. Klinikern oft unbekannt und eine enge Kollaboration mit den HLA-Laboren unabdinglich. Um dem Problem fehlender DSA-Information Rechnung zu tragen, wurde eine Ausweichmöglichkeit eingeräumt, indem gegebenenfalls mögliche Differentialdiagnosen im Befund vom Pathologen angegeben werden können.

Die neu auftretende arterielle Intimafibrose und die Inklusion der gegen nicht HLA-Antigene gerichteten Antikörper wurden für die DSA-Diagnostik komplett aus der Kategorie 2 entfernt [21]. Ursachen waren für Ersteres nicht fehlende Spezifität, sondern der ohne sequenzielle Biopsien problematische Nachweis und für Zweiteres fehlende einheitliche Nachweismethoden und eine bemerkenswerte Breite an potenziellen Antigenen.

## Validierungsbedarf molekularpathologischer Untersuchungen

Wesentliche Einschränkungen der Genexpressionsanalyse bleiben ihre eingeschränkte Verfügbarkeit und ihr Preis. Weiterhin müssen Indikationen solcher Analysen, welche immunologische Situation und welche technischen Voraussetzungen (welches Gerät, welche Transkriptsignaturen und welche Schwellenwerte) geklärt werden, um diese Technik breitflächiger anzuwenden. Die Banff Foundation hat bereits molekularpathologische Signaturen verschiedener Prozesse im Internet bereitgestellt [8]. Der erhebliche Wissensbedarf um diese Technologie wurde trotzdem während des letzten Banff-Meetings erneut betont. Rezente Studien – auch aus unserem Zentrum – haben relevante diagnostische und prognostische Daten im AMR-Kontext geliefert [5, 9, 12], wogegen überzeugende transkriptionelle Daten für die Diagnose der T‑Zellen-mediierten Abstoßung (TCMR) oder anderer Situationen wie z. B. Polyomavirusinfektion oder Pyelonephritis fehlen. Insgesamt muss der Nutzen, nicht nur für die Patientenbetreuung, sondern auch für die Finanzen des Gesundheitssystems, noch validiert werden. Aus diesen Gründen wurde die Formulierung dieses Punkts wie folgt ergänzt: „sofern für diesen Nutzungskontext gründlich validiert und verfügbar“.

## Banff-Arbeitsplan

Erwähnenswert sind, neben den Fortschritten in der AMR-Diagnostik, weitere lebhafte Besprechungen über Graft-Läsionen, insbesondere die chronisch-aktive TCMR oder Borderline-Läsionen, die Einführung von Aktivitäts- und Chronizitätsindexen und Lösungen für eine Software-basierte Klassifizierung in einem zweiten Manuskript [18].

## Fazit für die Praxis

- Basierend auf aktuellen wissenschaftlichen Arbeiten und aussagekräftigen Ergebnissen um die Pathophysiologie der antikörpermediierten Abstoßung (AMR) wurde die Kategorie der humoralen Abstoßung im Rahmen der letzten Banff-Konferenz aktualisiert.
- Der neue diagnostische Algorithmus empfiehlt den sequentiellen Einschluss verschiedener Informationen, zunächst der pathologischen Evaluation einer eventuellen mikrovaskulären Entzündung in der Transplantatbiopsie, dann der C4d- und DSA-Befunde (donorspezifische Antikörper), um einen der drei Abstoßungsphänotypen dieser Kategorie festzuhalten.
- Daraus resultieren neben dem klassischen Bild einer AMR, eine wahrscheinliche AMR-Befundkonstellation und ein deskriptiver Phänotyp „MVI („microvascular inflammation“), C4d-negativ, DSA-negativ“.
- Ziel ist die Situation akademischer Unwissenheit um den letzteren Phänotyp darzustellen und gleichzeitig der klinischen Routine gerecht zu werden.
- Die diagnostische Unterstützung durch molekularpathologische Daten wurde zwar befürwortet, muss aber aufgrund inkonstanter Verfügbarkeit und mangelnder Validierung noch evaluiert werden.
